# P-813. Combining an antibiotic stewardship program with 15-pathogen viral panel reduces inappropriate antibiotic prescribing in urgent care setting

**DOI:** 10.1093/ofid/ofaf695.1021

**Published:** 2026-01-11

**Authors:** Larissa May, Cosby G Arnold, Heejung Bang, Glenn Harnett, Tyra Furtado

**Affiliations:** University of California Davis, Sacramento, California; University of California, Davis, Sacramento, California; University of California Davis, Sacramento, California; No-Resistance Consulting Group, Birmingham, AL; University of California Davis, Sacramento, California

## Abstract

**Background:**

Acute upper respiratory tract infections (ARIs) frequently result in unnecessary antibiotic prescribing in outpatient settings, contributing to antibiotic resistance and avoidable adverse outcomes. While antimicrobial stewardship programs (ASPs) have shown promise, their implementation in urgent care settings remains limited. The impact of point-of-care (POC) respiratory viral panels on antibiotic prescribing is uncertain. This study evaluates the effect of the BIOFIRE® SPOTFIRE® Respiratory Panel, a 15-pathogen panel, with integrated ASP interventions on antibiotic prescribing in a high-volume urgent care setting in the Southeast United States.

**Figure:**
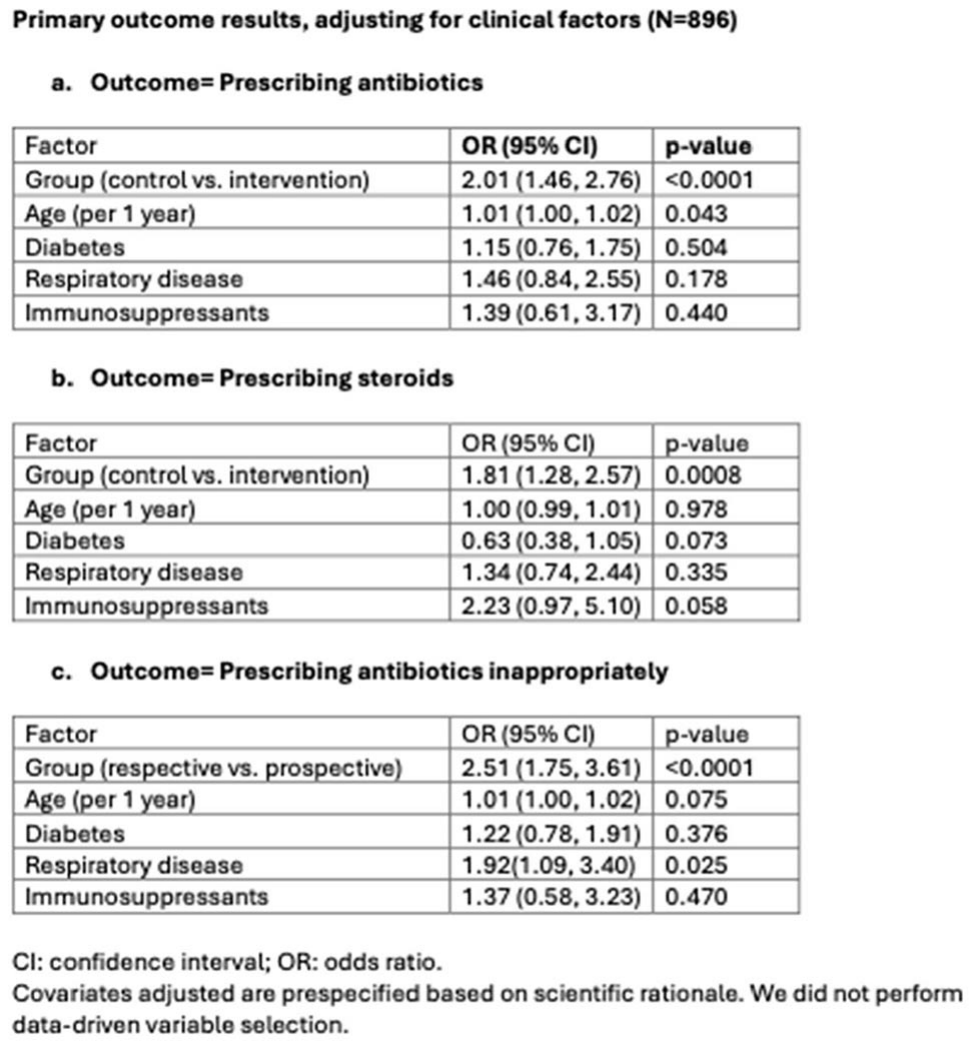

**Methods:**

We conducted a prospective cohort study of adults with ARI who presented to an urgent care in Louisiana from June to August 2024. The study was approved by a central Institutional Review Board. Patients were eligible for inclusion if they were 18 years of age or older and the urgent care provider planned to order a standard POC COVID-19 and/or influenza antigen test (consistent with a real-world study). We excluded patients who had a return visit within 72 hours or were unable to provide informed consent in English. A historic seasonally matched usual care group served as the control. This group was randomly selected from all patients seen for ARI, matched by month to control for seasonality, in the prior year. The intervention was the BIOFIRE® SPOTFIRE® Respiratory Panel testing and ASP.

**Results:**

A total of 296 patients were prospectively enrolled, with 600 randomly selected historical controls. The intervention group had a significantly lower antibiotic prescribing rate (24.3% vs. 38.2%; odds ratio (OR) 0.50, 95% confidence interval (CI) 0.36-0.68) and inappropriate antibiotic use rate (15.9% vs. 30.8%; OR 0.39, 95% CI 0.28-0.57) compared to controls. Steroid prescribing also decreased (17.9% vs. 29.0%; OR 0.55, 95% CI 0.39-0.78).

**Conclusion:**

Implementation of a POC multi-respiratory pathogen molecular test combined with an ASP intervention significantly reduced antibiotic and steroid prescribing in a high-volume urgent care setting.

**Disclosures:**

Larissa May, MD, MSPH, MSHS, Biomerieux: Advisor/Consultant|Biomerieux: Grant/Research Support|Biomerieux: Honoraria|Cytovale: Advisor/Consultant|GSK: Advisor/Consultant|Inflammatix: Advisor/Consultant|Inflammatix: Grant/Research Support|Inflammatix: Honoraria|Roche/Genmark: Advisor/Consultant|Roche/Genmark: Grant/Research Support|Roche/Genmark: Honoraria|Shiniogi: Advisor/Consultant|Thermofisher: Advisor/Consultant|Thermofisher: Honoraria Glenn Harnett, MD, BioMerieux: Honoraria

